# Shrimp Oil Extracted from Shrimp Processing By-Product Is a Rich Source of Omega-3 Fatty Acids and Astaxanthin-Esters, and Reveals Potential Anti-Adipogenic Effects in 3T3-L1 Adipocytes

**DOI:** 10.3390/md19050259

**Published:** 2021-04-30

**Authors:** Indrayani Phadtare, Hitesh Vaidya, Kelly Hawboldt, Sukhinder Kaur Cheema

**Affiliations:** 1 Department of Biochemistry, Memorial University of Newfoundland, St. John’s, NL A1C 5S7, Canada; isphadtare@mun.ca (I.P.); hbv302@mun.ca (H.V.); 2 Faculty of Engineering and Applied Science, Memorial University of Newfoundland, St. John’s, NL A1C 5S7, Canada; khawboldt@mun.ca

**Keywords:** 3T3-L1 adipocytes, astaxanthin, adipogenesis, shrimp processing by-product

## Abstract

The province of Newfoundland and Labrador, Canada, generates tons of shrimp processing by-product every year. Shrimp contains omega (n)-3 polyunsaturated fatty acids (PUFA) and astaxanthin (Astx), a potent antioxidant that exists in either free or esterified form (Astx-E). In this study, shrimp oil (SO) was extracted from the shrimp processing by-product using the Soxhlet method (hexane:acetone 2:3). The extracted SO was rich in phospholipids, n-3 PUFA, and Astx-E. The 3T3-L1 preadipocytes were differentiated to mature adipocytes in the presence or absence of various treatments for 8 days. The effects of SO were then investigated on fat accumulation, and the mRNA expression of genes involved in adipogenesis and lipogenesis in 3T3-L1 cells. The effects of fish oil (FO), in combination with Astx-E, on fat accumulation, and the mRNA expression of genes involved in adipogenesis and lipogenesis were also investigated. The SO decreased fat accumulation, compared to untreated cells, which coincided with lower mRNA expression of adipogenic and lipogenic genes. However, FO and FO + Astx-E increased fat accumulation, along with increased mRNA expression of adipogenic and lipogenic genes, and glucose transporter type 4 (*Glut-4*), compared to untreated cells. These findings have demonstrated that the SO is a rich source of n-3 PUFA and Astx-E, and has the potential to elicit anti-adipogenic effects. Moreover, the SO and FO appear to regulate adipogenesis and lipogenesis via independent pathways in 3T3-L1 cells.

## 1. Introduction

Obesity is a chronic medical condition and a major public health concern that is increasing in prevalence throughout the world. One in four adult Canadians or about 6.3 million people, were obese in 2011–2012 [1]. In 2018, 26.8% of Canadians 18 years of age and older (roughly 7.3 million adults) were reported as obese [2]. Newfoundland and Labrador (NL) has the highest rate of obesity in all of Canada [1,2]. Individuals with obesity are at a greater risk of chronic diseases such as cardiovascular diseases, dyslipidemia, hypertension, and diabetes [3,4,5]. Obesity is linked to changes in adipocytes function, thereby increasing the adipose tissue mass and size [6]. Adipocyte dysfunction is associated with insulin resistance, and alterations in the secretory function of adipocytes that lead to chronic low-grade inflammation [7].

The primary function of adipose tissue is to store energy by accumulating triacylglycerols (TAG) [8]. Adipose tissue is involved in regulating lipid metabolism, insulin-regulated glucose uptake, and inflammatory response [9,10]. Adipose tissue has substantial capacity to expand, which is evident by the storage of TAG in adipocytes resulting in the expansion of the adipose tissue (hypertrophy) [11,12,13] or adipocyte hyperplasia (increase in number). There are intricate sequences of events that regulate the TAG pool within the adipocyte such as de novo synthesis from carbon precursors called lipogenesis, and the process of TAG hydrolysis called lipolysis [14]. The process of differentiation of committed fibroblast-like preadipocytes into the lipid-laden adipocytes defines the process of adipogenesis [8,10].

A crucial step towards adipocyte differentiation involved the activation of regulatory genes such as, CCAAT enhancer-binding protein (C/EBP)-β, which is necessary for activation of peroxisome proliferator-activated receptor-γ (*Pparγ*) and C/EBPα that regulates adipocyte differentiation [15]. The regulation of lipogenesis in adipocytes involves another important transcription factor called sterol regulatory element-binding protein (SREBP)-1c [16], which promotes adipocyte differentiation by regulating the expression of genes linked to fatty acids and cholesterol synthesis [17]. Genes such as acetyl-CoA carboxylase (ACC1), fatty acid synthase (FASN), stearoyl-CoA desaturase (SCD1), and diacylglycerol acyltransferase (DGAT2) are regulated by SREBP-1c [18].

Omega (n)-3 polyunsaturated fatty acids (PUFA) have been shown to regulate adipocyte function, and to improve insulin resistance [19,20,21,22,23]. Although fish and fish oil supplements are the popular choices for n-3 PUFA supplements, Northern shrimp (*Pandalus borealis*), a cold-water shrimp found in the North Atlantic, is another popular marine source. Northern shrimp contains a high content of n-3 PUFA, and is rich in antioxidants [24], with a potential to provide health benefits [25]. The majority of the lipids in shrimp are present in the head and tail, however, these parts are commonly discarded as a waste material at shrimp processing units. The lack of information and data towards the nutritional value and health potential, along with the lack of regulation of ocean discharge for the fish processing plant, leads to shrimp heads and tails being discarded as waste. These body parts represent around 45–60% of the whole shrimp [26,27]. Commercially, shrimp are also separated by size, and the small size that does not meet the selling scale gets discarded. Since 2010, the total global capture stated for the Northern shrimp species is between 315,511–446,909 tons [28]. This potentially translates to ~150,000–220,000 tons of by-product discharged to the ocean. This leads to both, a loss of value, as well as detrimental environmental impact in the form of increased organic load on the ocean.

The shrimp processing by-product is a rich source of astaxanthin (Astx) and lipids [29]. Astx (3,3′-dihydroxy-beta, beta-carotene-4,4′-dione) is a xanthophyll carotenoid [30] that exists in either free form, conjugated with protein or esterified with one or two fatty acids, i.e., monoester or diester form, which stabilizes the molecule [31]. The antioxidant potential of Astx is suggested to be 10 times higher than other carotenoids, and Astx is an almost 100 times more potent antioxidant than vitamin E and C [30]. Esterified astaxanthin (Astx-E) has higher antioxidant activity, compared to free Astx [32]. In 2010, Astx extracted from *H. pluvialis* received the “generally recognized as safe (GRAS)” status from the US Food and Drug Administration (FDA) [33]. Astx is reported to reduce body weight gain, decrease hepatic and plasma total cholesterol and TAG levels, and improve insulin sensitivity in mice fed obesity-promoting diet [34], however, the Astx form (free/Astx-E) was not specified.

Different methods have been used to extract shrimp oil (SO) from shrimp [27,30,35,36], however, there is very little information in the literature on the methods to extract quality shrimp oil from shrimp processing by-product. Furthermore, there are no reports to date to establish the effects of SO extracted from shrimp processing by-product on the process of adipogenesis and lipogenesis. In the present study, we extracted SO from shrimp processing by-product using the Soxhlet method, and studied the lipids and Astx composition of shrimp extract. Then, we investigated the effects of shrimp extract (SE) and SO on fat accumulation, adipogenesis, and lipogenesis in 3T3-L1 adipocytes. Furthermore, we investigated whether a combination of fish oil (FO) plus Astx-E will reduce fat accumulation and inhibit adipogenesis to a greater extent, compared to FO alone. Our findings are the first to show the potential of shrimp processing by-product as a meaningful resource to develop nutraceuticals with anti-obesity properties.

## 2. Results

### 2.1. Lipid Composition of SO

The total lipids content was 3.92 mg/g wet shrimp processing by-product. The lipid composition showed that SO is rich in phospholipids (64.20 wt%) (Table 1). Other lipid components included TAG (13.66 wt%), alcohols (4.46 wt%), sterols (21.18 wt%), wax/steryl esters (0.67 wt%), methyl esters (0.56 wt%), ethyl esters (1.32 wt%), and free fatty acids (0.33 wt%).

### 2.2. Fatty Acids Composition of Oils

SO contained 18.33% (nmol/nmol) SFA, 40.58% (nmol/nmol) MUFA, and 41.08% (nmol/nmol) PUFA (Table 2). SFA comprised mainly of C16:0 (15.73% (nmol/nmol)) and C18:0 (2.42% (nmol/nmol)), while MUFA comprised mainly of C16:1n7 (9.58% (nmol/nmol)), C18:1n9 (21.33% (nmol/nmol)), and C18:1n7 (6.49% (nmol/nmol)). SO was high in n-3 PUFA (37.09% (nmol/nmol)), of which 21% (nmol/nmol) was EPA (C20:5n3) and 13.89% (nmol/nmol) was DHA (C22:6n3). SO contained a small amount of total n-6 PUFA (3.99% (nmol/nmol)), mainly comprising of C18:2n6 (1.96% (nmol/nmol)) and C20:4n6 (1.69% (nmol/nmol)).

FO contained 23.85% (nmol/nmol) SFA, 32.82% (nmol/nmol) MUFA, and 43.32% (nmol/nmol) PUFA (Table 2). SFA comprised of C16:0 (21.03% (nmol/nmol)) and C18:0 (2.73% (nmol/nmol)), while MUFA comprised mainly of C16:1n7 (18.30% (nmol/nmol)) and C18:1 (14.26% (nmol/nmol)). FO was high in n-3 PUFA (38.88% (nmol/nmol)), of which 20.14% (nmol/nmol) was EPA (C20:5n3) and 13.77% (nmol/nmol) was DHA (C22:6n3). FO contained a small amount of total n-6 PUFA (4.44% (nmol/nmol)), mainly comprising of C18:2n6 (1.95% (nmol/nmol)) and C20:4n6 (1.62% (nmol/nmol)).

### 2.3. Astaxanthin Content of SO

The TLC analysis confirmed the presence of Astx in SO, along with other unknown carotenoids (data not shown). Soxhlet extraction recovered 4.38 mL shrimp extract/g of shrimp processing by-product. Yields of free Astx and Astx-E were 24.03 and 187.76 μg/g of shrimp processing by-product, respectively (Table 3). Thus, the Astx-E content was almost 8 times higher than the free Astx.

### 2.4. SO Reduced Fat Accumulation in 3T3-L1 Adipocytes

**Figure 1:**
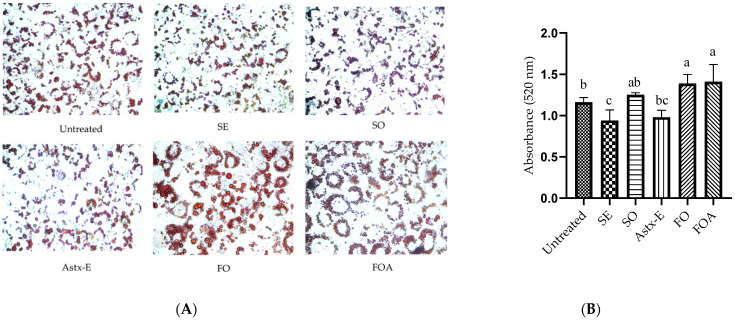
Shrimp extract decreased, while fish oil increased fat accumulation in 3T3-L1 mature adipocytes. Preadipocytes were differentiated to mature adipocytes in the presence or absence of various treatments for 8 days, and Oil Red O staining and quantification was performed as explained in the Methods section. (**A**) Representative images of the cells stained with Oil Red O on day 8 (400× magnification); (**B**) fat accumulation measured spectrophotometrically. Values are expressed as mean ± SD, *n* = 3. Data were analyzed using one-way ANOVA and Tukey’s post-hoc test; *p* < 0.05 was considered significant. Superscripts (a, b, c) represent significant differences. Untreated: Untreated cells; SE: Shrimp extract; SO: Shrimp oil; Astx-E: Esterified astaxanthin; FO: Fish oil; FOA: FO + Astx-E. Untreated vs. SE (*p* = 0.01); untreated vs. FO (*p* = 0.01); untreated vs. FOA (*p* = 0.01).

Preadipocytes were differentiated to mature adipocytes in the presence or absence of various treatments for 8 days. Oil Red O staining of SO, SE, and Astx-E treated cells revealed a decrease in fat accumulation, compared to untreated cells, while FO and FO + Astx-E (FOA) showed an increase in fat accumulation, compared to untreated cells (Figure 1A). Quantification of extracted Oil Red O confirmed a significant decrease (*p* = 0.01) in fat accumulation with SE, while FO and FOA significantly increased (*p* = 0.01) fat accumulation, compared to untreated cells (Figure 1B). Interestingly, quantification of extracted Oil Red O showed no significant effect on fat accumulation after treatment with SO and Astx-E, compared to control cells, even though Oil Red O staining revealed a decrease in fat accumulation.

### 2.5. Shrimp Oil Decreased, While Fish Oil Increased the mRNA Expression of Pparγ and Srebp1c in Mature 3T3-L1 Adipocytes

**Figure 2:**
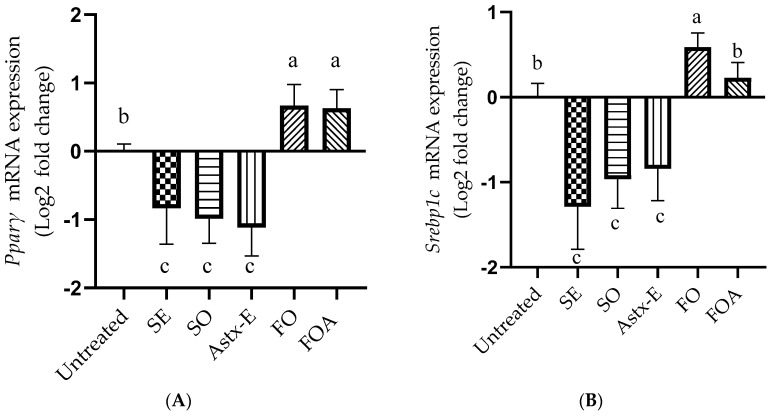
SO decreased, while FO increased the mRNA expression of *Pparγ* and *Srebp1c* in 3T3-L1 mature adipocytes. The cells were differentiated to mature adipocytes in the presence or absence of various treatments for 8 days, and the mRNA expression of **(A)** peroxisome proliferator-activated receptor (*Pparγ*), and **(B)** sterol regulatory element-binding protein (*Srebp1c*) was measured as explained in the Methods section. The mRNA expression was normalized to RPLP0 as the reference gene, and expressed as Log2 fold change. Data were analyzed using one-way ANOVA and Tukey’s post-hoc test; *p* < 0.05 was considered significant. Superscripts (a, b, c) represent significant differences, *n* = 3. Untreated: Untreated cells; SE: Shrimp extract; SO: Shrimp oil; Astx-E: Esterified astaxanthin; FO: Fish oil; FOA = FO + Astx-E. *Pparγ*, untreated vs. SO (*p* = 0.0006); untreated vs. SE (*p* = 0.004); untreated vs. Astx-E (*p* = 0.0001); untreated vs. FO (*p* = 0.01); untreated vs. FOA (*p* = 0.01). *Srebp1c*, untreated vs. SO (*p* = 0.0004); untreated vs. SE (*p* < 0.0001); untreated vs. Astx-E (*p* = 0.001); untreated vs. FO (*p* = 0.01); FO vs. SO (*p* < 0.0001); FO vs. SE (*p* < 0.0001); FO vs. Astx-E (*p* < 0.0001).

The mRNA expression of *Pparγ* decreased significantly in 3T3-L1 mature adipocytes after treatment with SO (*p* = 0.0006), SE (*p* = 0.004), and Astx-E (*p* = 0.0001), compared to untreated cells (Figure 2A). However, the mRNA expression of *Pparγ* increased significantly after treatment with FO (*p* = 0.01) and FOA (*p* = 0.01). Both FO and FOA had higher mRNA expression of *Pparγ*, compared to SO, SE, and Astx-E (*p* < 0.05). There was no effect of respective doses of the vehicle controls (PC, DMSO, PC + DMSO) on the mRNA expression of *Pparγ*, compared to the untreated cells (Supplementary Figure S1A).

The mRNA expression of *Srebp1c* decreased significantly in 3T3-L1 mature adipocytes after treatment with SO (*p* = 0.0004), SE (*p* < 0.0001), and Astx-E (*p* = 0.001), compared to untreated cells (Figure 2B). The treatment with SO, SE, and Astx-E also revealed significantly lower (*p* < 0.0001) mRNA expression of *Srebp1c*, compared to FO. However, the mRNA expression of *Srebp1c* was significantly higher after treatment with FO (*p* = 0.01), compared to untreated cells. There was no statistical difference between the cells treated with FOA and untreated cells, however, FOA revealed lower (*p* < 0.05) mRNA expression of *Srebp1c*, compared to FO. There was no effect of respective doses of the vehicle controls (PC, DMSO, PC + DMSO) on the mRNA expression of *Srebp1c*, compared to the untreated cells (Supplementary Figure S1B).

### 2.6. SO Decreased, While FO Increased the mRNA Expression of Lipogenic Genes and Glut-4 in Mature 3T3-L1 Adipocytes

**Figure 3:**
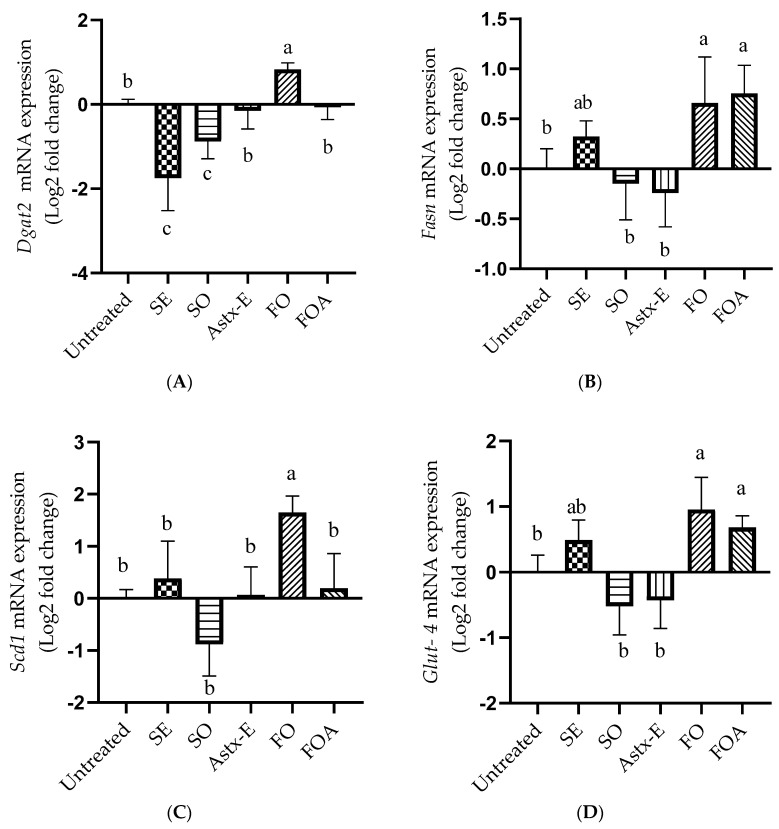
SO decreased, while FO increased the mRNA expression of lipogenic genes in mature 3T3-L1 adipocytes. The cells were differentiated to mature adipocytes in the presence or absence of various treatments for 8 days, and the mRNA expression analysis of (**A**) diacylglycerol O-acyltransferase 2 (*Dgat2*), (**B**) fatty acid synthase (*Fasn*), (**C**) stearoyl-coA desaturase-1 (*Scd1*), and (**D**) glucose transporter type-4 (*Glut-4*) was performed. Gene expression of the target genes was normalized to RPLP0 as the reference gene, and expressed as Log2 fold change. Data were analyzed using one-way ANOVA and Tukey’s post-hoc test; *p* < 0.05 was considered significant. Superscripts (a, b, c) represent significant differences, *n* = 3. Untreated: Untreated cells; SE: Shrimp extract; SO: Shrimp oil; Astx-E: Esterified astaxanthin; FO: Fish oil; FOA = FO + Astx-E. *Dgat2*, untreated vs. SO (*p* = 0.01); untreated vs. SE (*p* < 0.0001); untreated vs. FO (*p* = 0.01); FO vs. SO (*p* < 0.0001); FO vs. SE (*p* < 0.0001); FO vs. FOA (*p* = 0.01). *Fasn*, untreated vs. FO (*p* = 0.01); untreated vs. FOA (*p* = 0.007). *Scd1*, untreated vs. FO (*p* = 0.0009); FO vs. FOA (*p* = 0.003). *Glut-4*, untreated vs. FO (*p* = 0.0003); untreated vs. FOA (*p* = 0.01); SO vs. FO (*p* < 0.0001); SO vs. FOA (*p* < 0.0001); Astx-E vs. FO (*p* < 0.0001); Astx-E vs. FOA (*p* < 0.0001).

The mRNA expression of *Dgat2* was significantly lower after treatment with SO (*p* = 0.01) and SE (*p* < 0.0001), compared to untreated cells (Figure 3A). Furthermore, the *Dgat2* mRNA expression was significantly lower after treatment with SO and SE (*p* < 0.0001), compared to FO. There was no effect of Astx-E treatment on the expression of *Dgat2*, compared to untreated cells. Interestingly, the FO treatment showed significantly higher (*p* < 0.05) mRNA expression of *Dgat2*, compared to untreated cells. There was no statistical difference between the cells treated with FOA and untreated cells, however, the treatment with FOA showed significantly lower (*p* < 0.05) mRNA expression of *Dgat2*, compared to FO. There was no effect of respective doses of the vehicle controls (PC, DMSO, PC + DMSO) on the mRNA expression of *Dgat2*, compared to the untreated cells (Supplementary Figure S1C).

There was no significant effect of SO, SE, and Astx-E on the mRNA expression of *Fasn*, compared to untreated cells (Figure 3B). However, FO (*p* = 0.01) and FOA (*p* = 0.007) significantly increased the mRNA expression of *Fasn*, compared to untreated cells (Figure 3B). There was no effect of respective doses of the vehicle controls (PC, DMSO, PC + DMSO) on the mRNA expression of *Fasn*, compared to the untreated cells (Supplementary Figure S1D).

The treatment with SO, SE, and Astx-E had no significant effect on the mRNA expression of *Scd1*, compared to untreated cells (Figure 3C). Interestingly, the treatment with FO significantly increased (*p* = 0.0009) the mRNA expression of *Scd1*, compared to untreated cells. There was no statistical difference between the cells treated with FOA and untreated cells, however, *Scd1* mRNA expression was lower in the FOA (*p* = 0.003) treated cells, compared to FO. There was no effect of respective doses of the vehicle controls (PC, DMSO, PC + DMSO) on the mRNA expression of *Scd1*, compared to the untreated cells (Supplementary Figure S1E).

The treatment with SE, SO, and Astx-E showed no significant effect on the mRNA expression of *Glut-4*, compared to untreated cells (Figure 3D). FO (*p* = 0.0003) and FOA (*p* = 0.01) showed higher mRNA expression of *Glut-4*, compared to untreated cells. SO and Astx-E revealed lower mRNA expression of *Glut-4* (*p* < 0.05), compared to both FO and FOA (Figure 3D). There was no effect of respective doses of the vehicle controls (PC, DMSO, PC + DMSO) on the mRNA expression of *Glut-4*, compared to the untreated cells (Supplementary Figure S1F).

## 3. Discussion

In this study, we have demonstrated that SO extracted from shrimp processing by-product is a rich source of n-3 PUFA, phospholipids, and Astx-E. Furthermore, the treatment of 3T3-L1 cells with SO and SE extracted from shrimp processing by-product reduced fat accumulation, and showed a lower mRNA expression of genes involved in adipogenesis and lipogenesis. Interestingly, FO, alone or in combination with Astx-E, increased fat accumulation, and had a higher mRNA expression of genes involved in adipogenesis and lipogenesis. Previously, our laboratory has shown that marine species, such as blue mussels and sea cucumber that are rich in n-3 PUFA and other bioactives, reduced fat accumulation and decreased the mRNA expression of adipogenic and lipogenic genes in 3T3-L1 adipocytes with a potential to elicit anti-obesity effects [37,38]. The effects of SO and SE were similar to our previous observations. Furthermore, Shikov et al. [39] showed that lipids extracted from sea urchins body wall inhibit MAPK p38, cyclooxygenase COX-1, and COX-2, and suggested that these lipid fractions can be used towards drug development with anti-inflammatory activity. It is likely that the shrimp extract has anti-inflammatory properties, which needs to be tested in future studies.

Shrimp oil from shrimp processing by-product was rich in phospholipids (64.20 wt%). Previously, it has been shown that phospholipid-bound n-3 PUFA such as EPA (C20:5n3) and DHA (C22:6n3) are better absorbed due to better bioavailability, and therefore more efficiently delivered [40]. SO was also rich in n-3 PUFA, the amount of total n-3 PUFA was 37.09% nmol/nmol, with 21% EPA (C20:5n3) and 13.89% DHA (C22:6n3). A high proportion of phospholipids in SO extracted from shrimp processing by-product, along with a high amount of n-3 PUFA may suggest that these fatty acids are associated with phospholipids. SO extracted from shrimp processing by-product had a very small amount of free fatty acids (0.33 wt%). Generally, high free fatty acids cause hydrolytic rancidity and affect the quality of oils, thus, the amount of free fatty acids is considered as a quality parameter for oils [41]. The acceptable range of free fatty acids in commercially available krill oils is suggested to be up to 2 wt% [42], while the extracted SO contained much lower amounts of free fatty acids confirming superior quality.

The SE from shrimp processing by-product had significantly higher amounts of Astx-E, compared to free Astx. The Astx-E content was found to be almost 8 times higher than free Astx. It has been reported that Astx-E has a higher antioxidant activity compared to free Astx [32]. Thus, high amounts of Astx-E found in our shrimp extract compared to free Astx suggests it may have better health potential than free Astx. Overall, our findings suggest that the Soxhlet extraction method yields good quality oil from shrimp processing by-product. Early results show that the method of extraction impacts the distribution of lipids and yield of Astx in the final extract. While Soxhlet is a useful extraction method at the laboratory scale, scaling up is costly (economically and environmentally). Moreover, it has been stated that the use of edible oils as a “Green” solvent for extraction, and as a “Green” co-solvent in supercritical extraction can serve as an alternative to organic extractions [36]. Our results show that the “Greener” extraction methods have the potential for a higher quality product, however, the product needs to be tested in animal studies in the future.

N-3 PUFA and free Astx have independently been shown to reduce fat accumulation in 3T3-L1 cells [43,44,45]. However, there are no studies to date to show the effects of Astx-E on fat accumulation. Since shrimp processing by-product extracts (SE and SO) had high amounts of both n-3 PUFA and Astx-E, we investigated their effects on fat accumulation in 3T3-L1 cells. Preadipocytes were differentiated to mature adipocytes in the presence or absence of treatments for 8 days. SO and SE decreased fat accumulation, compared to untreated cells. On the other hand, FO and FO + Astx-E increased fat accumulation, compared to untreated cells. Interestingly, the Astx-E treatment reduced fat accumulation, while FO + Astx-E increased fat accumulation. These findings suggest a dominant effect of FO to regulate fat accumulation. Free Astx has been shown to reduce fat accumulation in 3T3-L1 adipocytes [45], which is similar to our findings with Astx-E.

Fat accumulation in the adipose tissue follows a sequential expression of genes involved in TAG synthesis and storage [46]. The crucial steps towards differentiation of preadipocytes into mature lipid laden adipocytes involves regulatory genes such as *Pparγ*, an important regulator of adipogenesis [47]. N-3 PUFA, such as EPA and DHA, and their metabolites are natural ligands for *Pparγ* [48], and act as antagonists. SO and SE from shrimp processing by-product decreased the mRNA expression of *Pparγ* significantly in mature adipocytes, compared to untreated cells. Furthermore, Astx-E also decreased the mRNA expression of *Pparγ*. On the other hand, FO and FO + Asx-E increased the mRNA expression of *Pparγ*, which coincided with an increase in fat accumulation. N-3 PUFA, such as EPA and DHA have generally been shown to decrease the mRNA expression of *Pparγ* in 3T3-L1 adipocytes [44,49,50]. However, we found an increase in *Pparγ* mRNA expression and fat accumulation after the treatment with FO. It is important to consider that we used FO to treat 3T3-L1 cells, and not pure n-3 PUFA, such as EPA or DHA. Although FO is rich in EPA and DHA, it contains other fatty acids and antioxidants that may exert a different effect on adipogenesis. That said, FO supplementation in animal studies have also been shown to reduce the mRNA expression of *Pparγ* [51]. However, there are no other studies to date to reveal the effects of FO and SO on adipogenesis in 3T3-L1 adipocytes. Previously, our laboratory has shown that arachidonic acid, an n-6 PUFA, has a dominant effect on the regulation of lipogenic genes when given along with EPA and DHA in 3T3-L1 adipocytes [52]. Therefore, it is possible that the effects observed with FO on adipogenesis are due to a combination of different fatty acids and other components in FO. Future studies will focus on animal studies to compare the effects of FO and SO on adipose tissue function.

The regulation of lipogenesis in adipocytes involves *Srebp1c*, another important transcription factor [16]. *Srebp1c* promotes adipocyte differentiation by regulating the expression of genes linked to fatty acid synthesis [17,53]. SO, SE, and Astx-E decreased the mRNA expression of *Srebp1c*, suggesting downregulation of lipogenesis. N-3 PUFA have been shown to reduce the mRNA expression of *Srebp1c* [54,55]. Interestingly, FO increased the mRNA expression of *Srebp1c*, while FO + Astx-E decreased the mRNA expression of *Srebp1c*, compared to FO. These findings suggest that Astx-E has a dominant effect on the regulation of *Srebp1c*, compared to FO. Previously, it has been reported that free Astx inhibited Akt activity, and reduced *Srebp1c* phosphorylation, which delayed nuclear translocation of *Srebp1c* and subsequent lipogenesis [56]. We did not measure phosphorylation or nuclear translocation of *Srebp1c* with various treatments. It is possible that the treatments affect post-transcriptional and post-translations modifications of *Srebp1c*, thereby affecting lipogenesis, which will be explored in the future.

Another key lipogenic gene that catalyzes the steps involved in the synthesis of palmitate (C16:0) from acetyl-CoA and malonyl-CoA is *Fasn* [57]. N-3 PUFA have been shown to downregulate the mRNA expression of *Fasn* in 3T3-L1 cells [50]. We found that SO, SE, and Astx-E had no significant effect on the mRNA expression of *Fasn*, compared to untreated cells. However, FO and FO+Astx-E revealed an increase in the mRNA expression of *Fasn*, compared to untreated cells. This coincides with an increase in fat accumulation after treatment with FO and FO+Astx-E, suggesting upregulation of lipogenesis. The synthesis of C16:0 by *Fasn* provides substrates for the synthesis of MUFA, specifically oleic acid (C18:1) from stearic acid (C18:0), a reaction catalyzed by *Scd1* [58]. SCD1 is the rate-limiting enzyme for the synthesis of MUFA from SFA, while MUFA are important for the synthesis of TAG [59]. N-3 PUFA reduce the mRNA expression of *Scd1* in 3T3-L1 adipocytes [44,50]. However, SO, SE, and Astx-E had no significant effect on the mRNA expression of *Scd1*. Although FO increased the mRNA expression of *Scd1*, FO + Astx-E had no effect. Furthermore, FO + Astx-E had lower mRNA expression of *Scd1*, compared to FO alone. These findings again suggest a dominant effect of Astx-E on the regulation of lipogenic genes, compared to FO. Our findings suggest that SO, FO, and Astx-E regulate lipogenesis via independent pathways.

*Dgat2* catalyzes the final step of mammalian TAG synthesis [60], thus, it is an important lipogenic gene responsible for fat accumulation in adipocytes. SO and SE reduced the mRNA expression of *Dgat2*, compared to untreated cells, which may be responsible for a decrease in fat accumulation. On the other hand, FO increased the mRNA expression of *Dgat2*, however, FO + Astx-E decreased the mRNA expression of *Dgat2*, compared to FO. This is similar to the effects of FO + Astx-E on *Srebp1c*, suggesting a dominant effect of Astx-E on *Dgat2* mRNA expression. Suppression of *Dgat2* is protective against excessive fat accumulation, obesity, and improved insulin resistance [61]. Our findings suggest that Astx-E may regulate *Dgat2* mRNA via *Srebp1c*. Since SO and SE had no significant effects on lipogenic genes, it is possible that the decrease in fat accumulation is via β-oxidation of fatty acids in 3T3-L1 adipocytes [62,63]. A recent study by Tsai et al. [64] showed that the effect of astaxanthin on fat accumulation and lipogenic genes is dose dependent. These authors found that a dose of 5 μg/mL of astaxanthin decreased fat accumulation, however, there was no effect on the mRNA expression of lipogenic genes. Increasing the astaxanthin dose to 25 μg/mL decreased fat accumulation, along with a decrease in lipogenic genes. It is possible that SO and FO have a dose dependent effect on fat accumulation and adipogenic and lipogenic gene expression, which will be explored in the future, along with measuring protein expression levels of the studied genes.

Fat accumulation in adipocytes is an essential process [65], however, excess fat accumulation predisposes cells towards insulin resistance [66]. Adipogenesis is associated with insulin sensitivity via insulin-mediated glucose uptake [67]. The increased uptake of glucose via GLUT-4 is positively linked with insulin sensitivity in adipose tissue [68]. SE, SO, and Astx-E had no significant effect on the mRNA expression of *Glut-4*, compared to untreated cells. On the other hand, FO and FO + Astx-E increased the mRNA expression of *Glut-4*. SO and Astx-E revealed lower mRNA expression of *Glut-4*, compared to both FO and FOA. In addition, we observed that FO and FO + Astx-E increased fat accumulation. Peyron-Caso et al. [69] reported that FO improves insulin sensitivity by regulating glucose transport as a result of increasing the GLUT-4 protein and mRNA levels in adipocytes of Sucrose-fed rats. It is likely that the effects of FO in 3T3-L1 adipocytes are associated with improving insulin sensitivity, whereas the effects of SE and SO are associated with the inhibition of adipogenesis and lipogenesis or increased beta-oxidation. Moreover, the effects of SO and SE were not always consistent, even though we used the same amount of SO and SE in our treatments. SE contained a lower amount of oil compared to SO, while SE also likely contained both water-soluble and insoluble components, due to the combination of polar and nonpolar solvent used for extraction. Further investigations are needed to understand the molecular mechanisms by which FO, SO, and SE regulate adipocyte function. Our findings demonstrate that the shrimp processing by-product is a valuable source for extracting bioactives, and for developing nutraceuticals with potential anti-obesity properties. Effective solutions to the problems of modern life are often found at the local level. Innovations which advocate approaches towards a circular economy are essential, as we seek to move towards a more sustainable future. This study intends to lay the groundwork for one such innovation. Such scientific approaches that encourage the concept of circular economy are especially pertinent to advance towards more sustainable ways of production and processing to develop novel nutraceuticals.

## 4. Materials and Methods

### 4.1. SO Extraction from Shrimp Processing By-Product

#### Soxhlet Method

The shrimp extract was prepared from the Northern shrimp (*Pandalus borealis*) processing by-product (provided by St. Anthony Basin Resources Inc. (SABRI), St. Anthony, NL, Canada). The Soxhlet method of extraction was used to extract lipids and carotenoids from the wet shrimp processing by-product (Supplementary Figure S2). The Northern shrimp (20 g) was ground to fine particles and placed in the thimble of the Soxhlet apparatus, to which 200 mL of hexane:acetone (2:3, *v*/*v*) was added and refluxed for 6 h. Four independent batches of extraction were performed. The extracts were pooled, concentrated in a rotatory evaporator (BUCHI Corporation, New Castle, DE, USA) at a temperature of 40 °C under a vacuum, and stored at 4 °C until further use. SO was extracted from the shrimp extract using the Folch method [70], and stored at 4 °C for future studies. Both the shrimp extract and SO were used for cell culture experiments.

### 4.2. Lipids and Fatty Acids Composition Analysis of Oils

The lipid composition of SO was analyzed using Thin Layer Chromatography-flame ionization detection (TLC-FID) (Mark VI Iatroscan, NTS, USA), and PeakSimple software (version 4.54, 6 channel, SRI Instruments, Torrance, CA, USA). The samples were spotted on silica coated chromarods, which were developed as per a previously established method [71,72]. Lipid classes for each chromarod were obtained using the regression equations derived during calibration to determine the percentage lipid composition (wt%), along with the total lipid content. The calibration was performed using a standard (Sigma Chemicals, St. Louis, MO, USA) with the composition: Nanodecane (hydrocarbons, 1.360 g/L), cholesteryl palmitate (wax ester/steryl esters, 1.060 g/L), 3-hexdecanone (ketones, 2.410 g/L), tripalmitin (triacylglycerol, 2.110 g/L), palmitic acid (free fatty acids, 1.030 g/L), cetyl alcohol (alcohol, 2.020 g/L), cholesterol (sterol, 1.480 g/L), monopalmitoyl (acetone mobile polar lipids, 2.070 g/L), phosphatidylcholine dipalmitoyl (phospholipids, 2.330 g/L). The calibration was performed by spotting two consecutive chromarods with 0.5, 1, 1.5, 2, and 3 μL of the standard. The correlation coefficients were attained by considering the amount of lipid extracts spotted on the chromarod of TLC-FID and the obtained peak area for each rod, while maintaining R2 values above 0.95 (correlation above 95%) for each lipid class.

Total lipids (SO and FO) were transmethylated, along with an internal standard (heptadecanoate, C17:0), and the fatty acids composition was analyzed using gas chromatography (GC)-FID (PerkinElmer, Waltham, MA, USA) as per our previously published method [73]. The concentration of each fatty acid was determined using the internal standard, and expressed as the percentage nmol of the total extracted fatty acids.

### 4.3. Astaxanthin Analysis

Astaxanthin from the shrimp extract was analyzed using TLC [74]. The shrimp extract was spotted on pre-coated Silica gel-G plates (# 805012; Macherey-Nagel, Düren, NRW, Germany), along with free Astx and Astx-E standards (Sigma-Aldrich, Oakville, ON, Canada; # SML0982 and # 1044210, respectively) and eluted using a mobile phase of acetone:hexane (25:75, *v*/*v*). The spots corresponding to each fraction were scraped, and collected in 250 μL of ethanol. Since astaxanthin is prone to oxidation, the procedure was undertaken with minimal exposure to light. The Astx content of each fraction was measured spectrophotometrically using respective standard curves for free and Astx-E standards (free Astx; # SML0982, Astx-E; # 1044210, Sigma-Aldrich, Canada) [74,75]. Calibration curves for free Astx were in the range of 2.5–40 μg/mL, and for esterified astaxanthin were 10–100 μg/mL. Ethanol was used as a blank. The absorbance was recorded at 472 nm using a spectrophotometer by following the method of Meyers and Bligh [74,75].

### 4.4. The 3T3-L1 Cell Culture

#### 4.4.1. Materials

The 3T3-L1 preadipocytes were obtained from American Type Culture Collection (ATCC, # CL-173, Manassas, VA, USA). Dulbecco’s Modified Eagle Medium (DMEM; # 41965039; Gibco Life Technologies, Burlington, ON, Canada), sodium pyruvate (100 mM; # 11360070; Gibco Life Technologies, Canada), newborn calf serum (NBCS; # 26010074; Gibco Life Technologies, Canada), and fetal bovine serum (FBS; # 12484028; Gibco Life Technologies, Canada). Insulin solution (10 mg/mL in 25 mM HEPES, pH 8.2; # I0516; Sigma-Aldrich, Canada), 3-isobutyl-1-methylxanthine (IBMX; # I5879; Sigma-Aldrich, Canada), and dexamethasone (Dex; # D4902; Sigma-Aldrich, Canada). L-α-phosphatidylcholine (PC; # P3556; Sigma-Aldrich, Canada), dimethyl sulfoxide (DMSO; # D2650; Sigma-Aldrich, Canada), Menhaden fish oil (FO; # F8020; Sigma-Aldrich, Canada), and glycerol (# G2025; Sigma-Aldrich, Canada).

#### 4.4.2. Culturing 3T3-L1 Cells

The 3T3-L1 preadipocytes were maintained in DMEM, containing 10% calf serum (NBCS) in a 5% CO_2_, humidified environment at 37 °C. Once the preadipocytes reached 70–80% confluency, differentiation was induced using the fresh medium containing DMEM + 10% FBS, insulin (10 μg/mL), IBMX (0.5 mM), and Dex (1 μM), and designated as Day 0 [52,76]. The differentiation medium was replaced with DMEM + 10% FBS and 10 μL/mL insulin after 48 h (day 2). Then, culture media were changed to fresh DMEM + 10% FBS on days 4 and 6 until day 8 of differentiation, with day 8 representing fully differentiated mature adipocytes.

#### 4.4.3. Preparation of Lipid Emulsions to Treat 3T3-L1 Cells

Lipid emulsions were prepared [77] to treat 3T3-L1 cells. Lipid emulsions were prepared using L-α-phosphatidylcholine (1.2%, *w*/*v*), glycerol (2.5%, *w*/*v*), fish oil or shrimp oil (10%, *w*/*v*), and autoclaved water, followed by ultrasonication using a modification to the method followed by Meisel et al. [78]. To prepare the oil emulsions, autoclaved water was slowly added to a mixture of PC and glycerol, followed by dropwise addition of the oil sample. The mixture was sonicated for 3 min with 60 s intervals using a 22.5 KHz sonicator (MicrosonTM, Model XL-2000, Ultrasonic liquid processor, Newtown, CT, USA). The samples were kept on ice during the entire process. Fresh lipid emulsions were prepared for all experiments.

The size distribution of emulsion particles was characterized using the dynamic light scattering (DLS) method [79] to confirm the uniform distribution of the lipid emulsion. DLS measurements were carried out using a Zetasizer 1000/3000 Hs (Malvern Instruments, Malvern, WR, UK), which uses a helium neon laser light and integrated analysis software. The temperature was adjusted to 25 °C, and the scattering angle was set to 90° before the measurements. The data were expressed as the z-average (d. nm) and polydispersity index (PDI). The size of the particles was in the nano-range with a Z-average of 0.61 nm (Supplementary Figure S3). The PDI value of 0.0 is considered as a perfectly uniform sample, whereas a value of 1.0 value is considered as a highly polydisperse sample with respect to the particle size [80]. The PDI of the emulsion particles was 0.16, indicating a relatively uniform (monodisperse) distribution of the particles.

#### 4.4.4. Pilot Study to Establish the Effects of Oil Emulsions on Cell Differentiation

In a pilot study, the effects of various concentrations of fish oil emulsions (0.25, 0.5, 0.75, 1 mg/mL culture medium) was investigated on adipogenesis in 3T3-L1 adipocytes. The 3T3-L1 preadipocytes were grown in a culture medium (DMEM + 10% NBCS) as described above until reaching 70–80% confluency. Differentiation was then induced as described above, in the presence or absence of lipid emulsions. The differentiation medium was replaced with DMEM + 10% FBS and 10 μL/mL insulin, along with treatments after 48 h (day 2). Then, culture media were changed to fresh DMEM + 10% FBS on days 4 and 6, in the absence or presence of treatments until day 8 of differentiation. The 3T3-L1 preadipocytes were also treated with various doses of PC (30, 60, 90, 120 μg/mL culture medium) that were used to prepare lipid emulsions. Untreated normal control cells received culture media only. On day 8, the cells were viewed using a Leica DMIL-LED Microscope (Leica Microsystems, Concord, ON, Canada) at 40× magnification, and the Infinity Camera Analyze Software (version 6.5.5, Lumenera Corporation, Ottawa, ON, Canada) was used for capturing the images (Supplementary Figure S4). Fish oil at a concentration of 0.25 mg/mL showed no adverse effects on 3T3-L1 cells until day 8 of differentiation, compared to control untreated cells (Supplementary Figure S4), while a 0.5 mg/mL concentration caused cell detachment by day 8, and the higher concentrations caused cell detachment within 3 days. Thus, future experiments were designed using 0.25 mg/mL of oil emulsions.

#### 4.4.5. Cell Metabolic Activity

Preadipocytes with a density of 1 × 10^4^ cells per well were seeded in 96-well microplates and incubated for 24 h. The culture medium was then replaced with fresh culture media (DMEM + 10% NBCS), along with different concentrations of the oil emulsions: SO, SE, and FO (0.125, 0.25, and 0.5 mg/mL culture medium). These concentrations of SO and SE contained 7.95, 15.9, and 31.8 ng Astx-E, respectively. Thus, cells were also treated with Astx-E (7.95, 15.9, and 31.8 ng/mL culture medium) and FO + Astx-E (0.125 mg + 7.95 ng, 0.25 mg + 15.9 ng, and 0.5 mg + 31.8 ng)/mL culture medium). The cells were also treated with various concentrations of PC that were used to prepare lipid emulsions, and DMSO to dissolve astaxanthin, and PC + DMSO. Untreated normal control cells received culture media only. The cells were incubated for 48 h, and then the MTT (3-(4,5-dimethylthiazol-2-yl)-2,5-diphenyltetrazolium bromide, a yellow tetrazole) colorimetric assay was performed to measure the cell metabolic activity [81]. The absorbance was recorded at 570 nm using a spectrophotometer, and the cell metabolic activity was calculated as a percentage fold change with respect to the untreated control cells. Vehicles used in the treatments (PC, DMSO, PC + DMSO) revealed no effect on the cell metabolic activity, compared to the untreated cells (Supplementary Figure S5A). Various concentrations of SE, SO, Astx-E, FO, and FO + Astx-E had no effect on the cell metabolic activity, compared to untreated cells (Supplementary Figure S5B).

#### 4.4.6. Treatments of 3T3-L1 Preadipocytes with Oil Emulsions to Measure Fat Accumulation, and the Expression of Adipogenic and Lipogenic Genes

**Figure 4:**
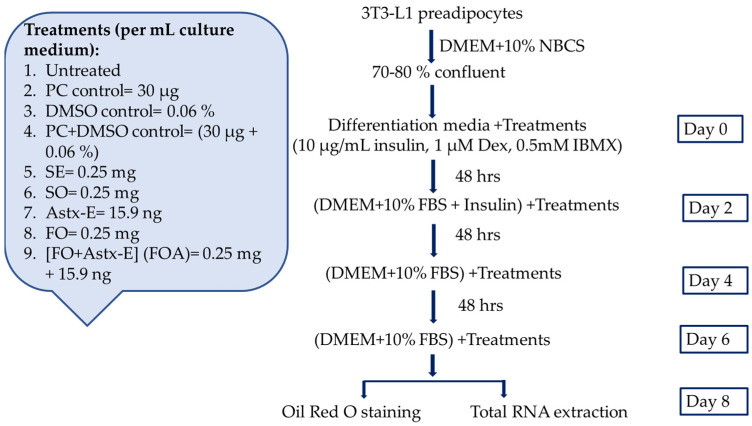
Experimental design to investigate the effects of shrimp extract, shrimp oil, and fish oil with or without Astx-E, on adipogenesis in 3T3-L1 cells. The 3T3-L1 preadipocytes were differentiated to mature adipocytes in the presence or absence of various treatments for 8 days, fat accumulation and the mRNA expression of genes involved in adipogenesis and lipogenesis were studied. Untreated: Untreated cells; PC: l-α-phosphatidylcholine; DMSO: Dimethyl sulfoxide; PC + DMSO: L-α-phosphatidylcholine + dimethyl sulfoxide; SE: Shrimp extract; SO: Shrimp oil; Astx-E: Esterified astaxanthin; FO: Fish oil; FOA: FO + Astx-E; DMEM: Dulbecco’s modified eagle medium; NBCS: Newborn calf serum; Dex: Dexamethasone; IBMX: 3-isobutyl-1-methylxanthine; FBS: Fetal bovine serum.

The 3T3-L1 preadipocytes were grown in a culture medium, and differentiated as described above in the presence or absence of treatments until day 8 of differentiation (Figure 4). Cells were differentiated to mature adipocytes in the presence or absence of SE, SO or FO at a final concentration of 0.25 mg/mL, for 8 days. This concentration of SO contained 15.9 ng Astx-E, thus, cells were also treated with 15.9 ng/mL of Astx-E, and FO plus 15.9 ng/mL of Astx-E. Cells also received the appropriate vehicles (30 µg/mL PC, 0.06% DMSO, and 30 μg/mL + 0.06% PC + DMSO). Fully differentiated cells were washed with 1× PBS and harvested to perform Oil Red O staining to study fat accumulation, and total RNA extraction to measure gene expression.

### 4.5. Oil Red O Staining

Lipid accumulation in 3T3-L1 adipocytes was measured using Oil Red O staining (# O1391, Sigma-Aldrich, Canada) as per the protocol. Oil Red O-stained cells were viewed using a Leica DMIL-LED Microscope at 400× magnification, and Infinity Camera Analyze Software (version 6.5.5) was used to capture the images. Oil Red O-dye was extracted from cells by adding isopropanol, the absorbance of extracted dye was measured using a spectrophotometer at 520 nm, and isopropanol was used as a blank.

### 4.6. Total RNA Extraction and Real-Time Quantitative Polymerase Chain Reaction

Total RNA was extracted from mature, fully differentiated 3T3-L1 adipocytes on day 8 using the TRIzol (Invitrogen, Carlsbad, CA, USA) method [82]. DNA contamination was removed using the DNase treatment (Promega, Madison, WI, USA). Nanodrop 2000 (Thermo Scientific, Waltham, MA, USA) was used to determine the concentration and purity (260/280) of extracted RNA, and RNA integrity was confirmed using 1.2% agarose gel. The gene expression analysis was performed using the Bio-Rad CFX96^TM^ Real-Time System. Primers used in the real-time quantitative polymerase chain reaction (qPCR) were designed using NCBI primer blast, and purchased from IDT Technologies (Coralville, IA, USA). The efficiency of all primers was within the acceptable range of 90–110%. Primer sequences are presented in Supplementary Table S1. Amplification was carried out using iQ SYBR Green Supermix (# 1708880, Bio-Rad, Hercules, CA, USA), and a reaction volume was 10 μL with 50 ng of cDNA per reaction as per our previous publications [83]. Data analyses were carried out using the CFX Manager TM Software, version 3.0 (Bio-Rad, Hercules, CA, USA). The delta Ct values for each gene of interest were obtained, and the mRNA expression of target genes was normalized to RPLP0 as the reference gene, a large ribosomal protein. The expression of target genes was calculated using the ΔΔCt method [84].

### 4.7. Statistical Analysis

The GraphPad Prism Software, version 8 (GraphPad Software, San Diego, CA, USA), was used for analyzing all data. Gene expression data were analyzed using one-way ANOVA, followed by Tukey’s post-hoc test. Results were expressed as mean ± standard deviation (SD), *n* = 3 (each experiment was repeated three times with three independent wells); *p* < 0.05 was considered significant. Superscripts (a, b, c) represent significant differences.

## 5. Conclusions

**Figure 5:**
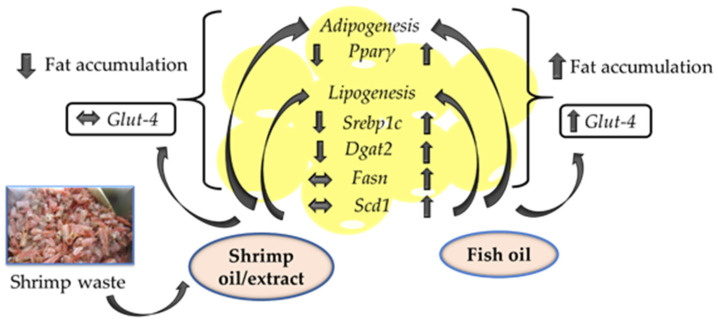
Schematic representation of the effects of shrimp oil/extract from shrimp processing by-product, and fish oil on the regulation of adipogenic and lipogenic genes in 3T3-L1 adipocytes. *Pparγ*: Peroxisome proliferator-activated receptor-gamma; *Srebp1c*: Sterol regulatory element-binding protein 1; *Dgat2*: Diacylglycerol O-acyltransferase 2; *Fasn*: Fatty acid synthase; *Scd1*: Stearoyl-CoA desaturase; *Glut-4*: Glucose transporter type 4. The up arrow indicates an increase in the mRNA expression of the respective gene; the down arrow indicates a decrease in the mRNA expression of the respective gene; the straight arrow indicates no change in the mRNA expression of the respective gene, compared to untreated cells.

Overall, we found that shrimp oil extracted from shrimp processing by-product using the Soxhlet extraction method reduced fat accumulation in 3T3-L1 cells, whereas fish oil increased fat accumulation. Interestingly, fish oil increased the mRNA expression of *Glut-4*, while shrimp oil showed no significant effect. Furthermore, shrimp oil had no significant effect on *Scd1* and *Fasn*, whereas fish oil increased it. We have proposed the pathways for the actions of shrimp oil and fish oil on adipogenesis and lipogenesis (Figure 5). It is likely that the effects observed with shrimp oil and fish oil on adipogenesis are not just due to the n-3 PUFA, but due to other fatty acids and components. Furthermore, different effects of shrimp oil and shrimp extract may also be due to the presence of Astx, along with other unknown carotenoids. It is clear that fish oil and shrimp oil regulate adipogenesis via independent pathways that need to be investigated in the future. Nonetheless, findings from this study have established a method to extract high quality shrimp oil from shrimp processing by-product, which can be used as a nutraceutical with potential anti-obesity properties.

## Figures and Tables

**Table 1:** Lipid composition of shrimp oil extracted from shrimp processing by-product.

Lipid Composition Wt (%)	Shrimp Oil
Wax/Steryl esters	0.67 ± 0.09
Methyl esters	0.56 ± 0.06
Ethyl esters	1.32 ± 0.11
Triacylglycerols	13.66 ± 5.35
Free fatty acids	0.33 ± 0.47
Alcohols	4.46 ± 0.36
Sterols	21.18 ± 1.69
Phospholipids	64.20 ± 4.68

Total lipids were extracted and analyzed as explained in the Methods section. Data are expressed as the percentage weight of the total extracted lipids, values are expressed as mean ± SD, *n* = 2.

**Table 2:** Fatty acids composition of shrimp oil extracted from shrimp processing by-product and fish oil.

Fatty Acids (% nmol/nmol)	Shrimp Oil	Fish Oil
C14:0	0.17 ± 0.00	0.09 ± 0.00
C16:0	15.73 ± 0.33	21.03 ± 0.06
C16:1n7	9.58 ± 0.65	18.30 ± 0.36
C18:0	2.42 ± 0.07	2.73 ± 1.13
C18:1n9	21.33 ± 1.03	5.95 ± 3.51
C18:1n7	6.49 ± 1.56	8.30 ± 5.78
C18:2n6	1.96 ± 0.12	1.95 ± 0.04
C18:3n6	0.30 ± 0.09	0.55 ± 0.01
C18:3n3	0.61 ± 0.08	1.69 ±0. 03
C20:1n9	0.45 ± 0.20	0.27 ±0. 00
C20:4n6	1.69 ± 0.14	1.62 ±0. 03
C20:5n3	21.10 ± 0.11	20.14 ± 0.4
C22:4n6	0.03 ± 0.05	0.33 ±0. 00
C22:5n3	1.48 ± 0.11	3.28 ±0. 07
C22:6n3	13.89 ± 0.13	13.77 ± 0.28
∑SFA	18.34 ± 0.25	23.85 ± 1.06
∑MUFA	40.58 ± 0.09	32.82 ± 1.89
∑n-3 PUFA	37.09 ± 0.04	38.88 ± 0.8
∑n-6 PUFA	3.99 ± 0.11	4.44 ± 0.03

The fatty acids composition of shrimp oil and fish oil was measured as described in the Methods section. Data are expressed as the percentage nmol of the total extracted fatty acids; values are expressed as mean ± SD, *n* = 2. ΣSFA: Sum of saturated fatty acids; ΣMUFA: Sum of monounsaturated fatty acids; ΣPUFA: Sum of polyunsaturated fatty acids; Σn-3 PUFA: Sum of omega-3 PUFA; Σn-6 PUFA: Sum of omega-6 PUFA.

**Table 3:** Astaxanthin content of shrimp extract.

Fraction	Concentration (μg/mL Shrimp Extract)	Astaxanthin Yield (μg/g Shrimp Processing By-Product)
Free Astx	8.24	24.03
Astx-E	64.37	187.76

The astaxanthin spots corresponding to each fraction were scrapped from TLC plates, and analyzed spectrophotometrically as mentioned in the Methods section. Free Astx: Free astaxanthin; Astx-E: Esterified astaxanthin.

## Data Availability

Not applicable.

## Supplementary Materials

The following are available online at https://www.mdpi.com/article/10.3390/md19050259/s1. Figure S1: The vehicle controls had no significant effect on the mRNA expression of *Pparγ*, *Srebp1c*, *Dgat2*, *Fasn*, *Scd1*, and *Glut-4*, compared to the untreated cells; Figure S2: Schematic of shrimp processing by-product extraction to prepare the shrimp extract/oil; Figure S3: Particle size analysis of the oil emulsions; Figure S4: Effects of fish oil on adipogenesis in 3T3-L1 adipocytes; Figure S5: Effects of lipid emulsions on the cell metabolic activity of 3T3-L1 preadipocytes; Table S1: Primer sequences for the real-time quantitative polymerase chain reaction.
