# Supplementary material for: Shrimp Oil Extracted from Shrimp Processing By-Product Is a Rich Source of Omega-3 Fatty Acids and Astaxanthin-Esters, and Reveals Potential Anti-Adipogenic Effects in 3T3-L1 Adipocytes
Source: Mar Drugs. 2021 Apr 30;19(5):259. doi: 10.3390/md19050259 (PMC8146821; doi:10.3390/md19050259)
Supplement: Supplementary file 1 [file marinedrugs-19-00259-s001.zip › marinedrugs-1198579-supplementary.pdf]

# Shrimp oil extracted from shrimp processing by-product is a rich source of omega-3 fatty acids and astaxanthin-esters, and reveals potential anti-adipogenic effects in 3T3-L1 adipocytes

**Indrayani Phadtare [1], Hitesh Vaidya [1], Kelly Hawboldt [2], and Sukhinder Kaur Cheema [1,*]**

[1] Department of Biochemistry, Memorial University of Newfoundland, St. John's, Newfoundland and Labrador, Canada A1B 3X9; isphadtare@mun.ca (I.P); hbv302@mun.ca (H.V); skaur@mun.ca (S.K.C)

[2] Faculty of Engineering and Applied Science, Memorial University of Newfoundland, St. John's, Newfoundland and Labrador, Canada A1B3X7; khawboldt@mun.ca (K.H)

*Correspondence: skaur@mun.ca (S.K.C);
Tel.: +1-709-864-3987 (S.K.C); Fax: +1-709-864-2422 (S.K.C)

**Supplementary data**

**Supplementary S1**

**Vehicle controls had no significant effect on the mRNA expression of *Pparγ, Srebp1c, Dgat2, Fasn, Scd1* and *Glut-4,* compared to the untreated cells.**

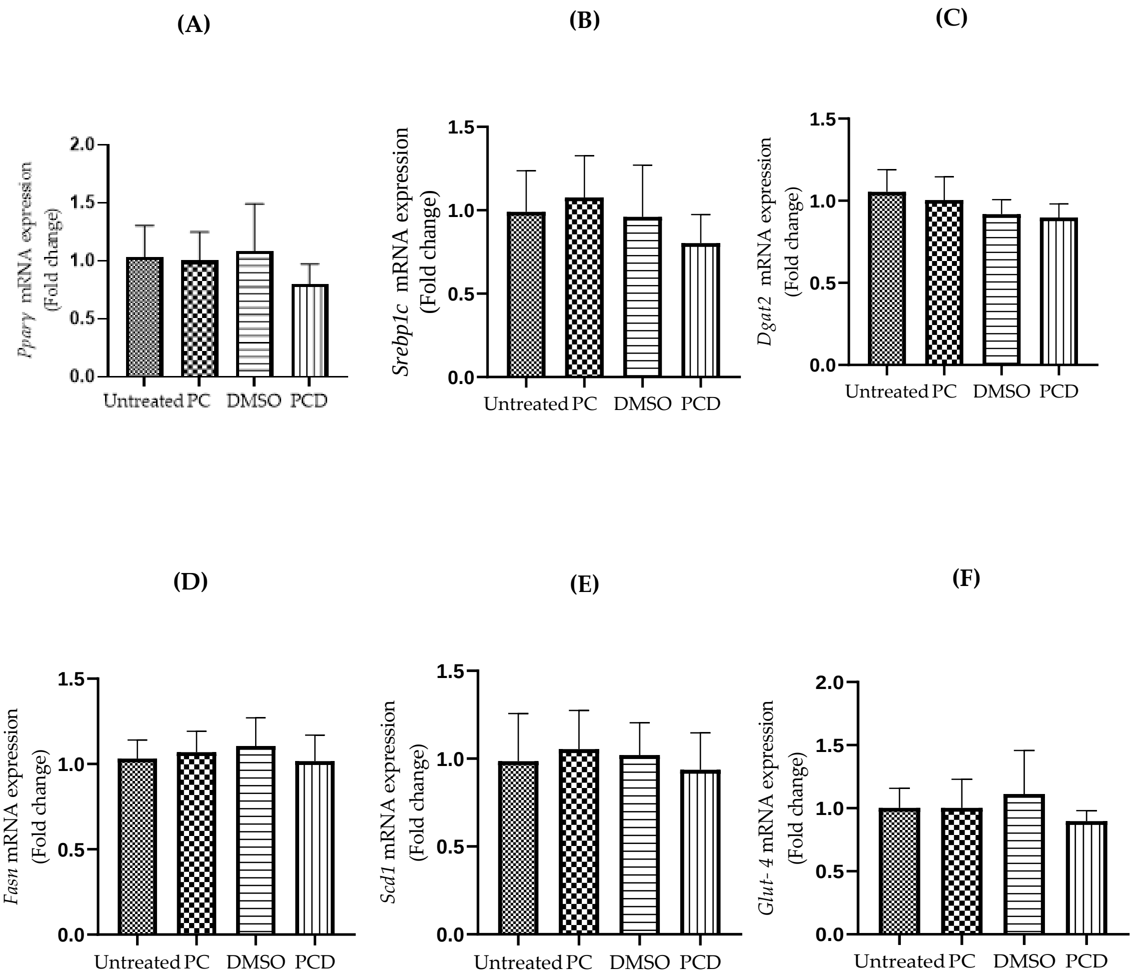


**Figure S1.** The cells were differentiated to mature adipocytes, for 8 days, as explained in the methods section. Total RNA was extracted, and the mRNA expression analysis of (**A**) Peroxisome proliferator-activated receptor (*Pparγ*), (**B**) sterol regulatory element-binding protein (*Srebp1c*), (**C**) diacylglycerol O-acyltransferase 2 (*Dgat2*), (**D**) Fatty acid synthase (*Fasn*), (**E**) Stearoyl-CoA desaturase-1 (*Scd1*), and (**F**) Glucose transporter type 4 (*Glut-4*) genes was performed. Expression of target genes was normalized to RPLP0 as the reference gene, and data were expressed as fold change. Vehicles: PC, DMSO, PCD. Data were analyzed using one-way ANOVA and Tukey's post-hoc test. $p<0.05$ was considered significant. n=3. Untreated=Untreated cells, PC=L-α-phosphatidylcholine (30 μg/mL culture medium), DMSO=dimethyl sulfoxide (0.06 %), PCD=PC+DMSO [(30 μg + 0.06 %)/mL culture medium].

**Supplementary S2**
**Schematic of shrimp processing by-product extraction to prepare shrimp extract and shrimp oil**

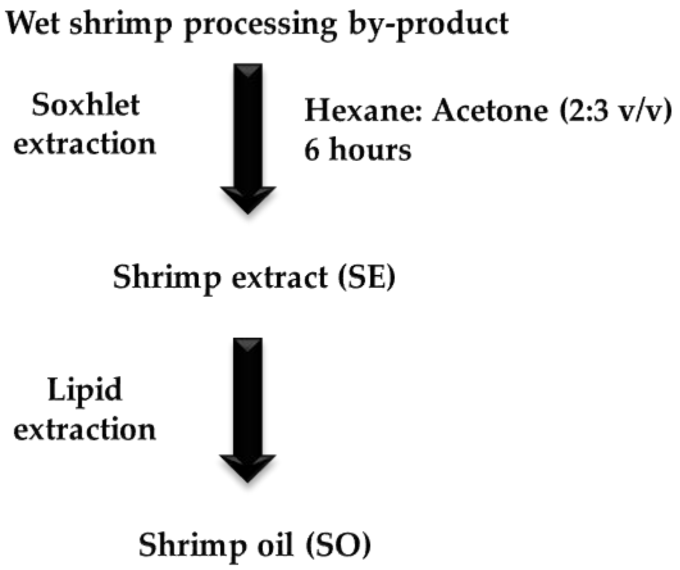


**Figure S2.** Schematic of shrimp processing by-product extraction to prepare shrimp extract and shrimp oil

**Supplementary S3**
**Particle size analysis of oil emulsions**

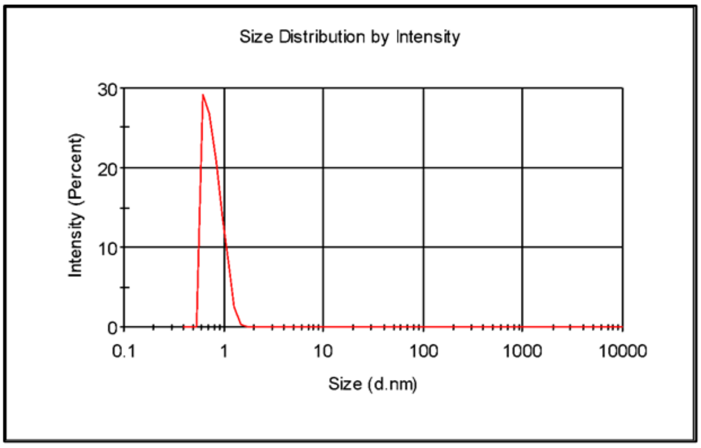


**Figure S3.** Analysis of particle size distribution of oil emulsions using dynamic light scattering (DLS). DLS measurements were carried out using a Zetasizer 1000Hs/3000Hs (Malvern Instruments, Worcestershire, U.K.) as described in the methods section. The data were expressed as the z-average (d. nm) and polydispersity index (PDI).

**Supplementary S4**
**Effect of fish oil on adipogenesis in 3T3-L1 adipocytes**

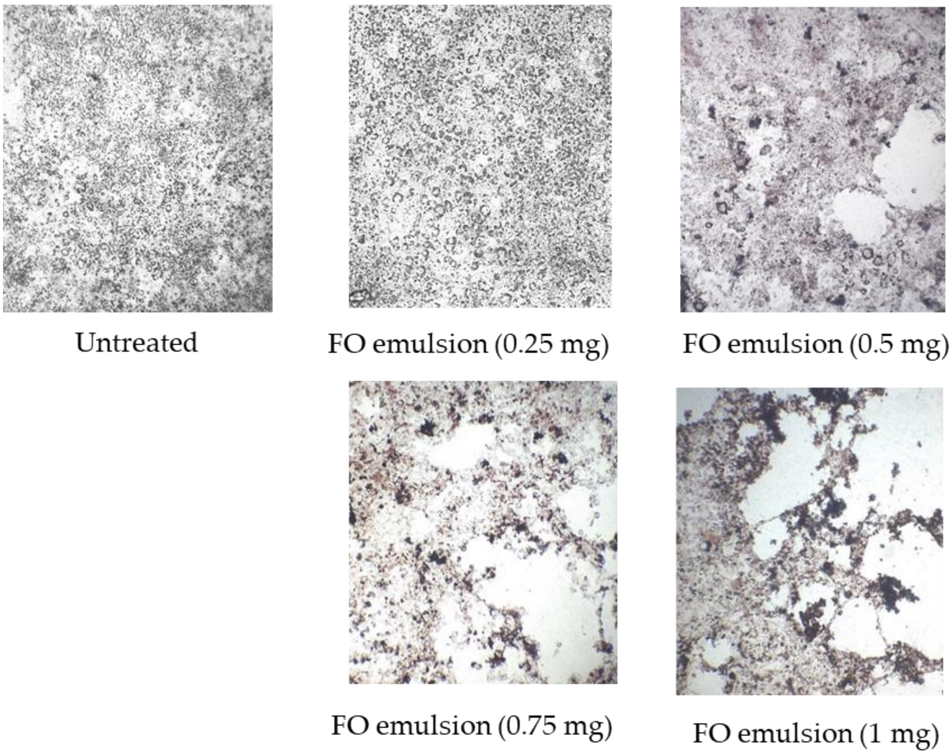


**Figure S4.** Preadipocytes were differentiated to mature adipocytes in the presence or absence of different concentrations of fish oil emulsions (0.25, 0.5, 0.75 and 1 mg/mL culture medium), for 8 days, as explained in the methods section. On day 8, the cells were viewed using a Leica DMIL LED Microscope at 40x magnification, and Infinity Camera Analyze Software (version 6.5.5) was used for capturing the images. Untreated=Untreated cells, FO=Fish oil.

**Supplementary S5**
**Effect of lipid emulsions on the cell metabolic activity of 3T3-L1 preadipocytes**

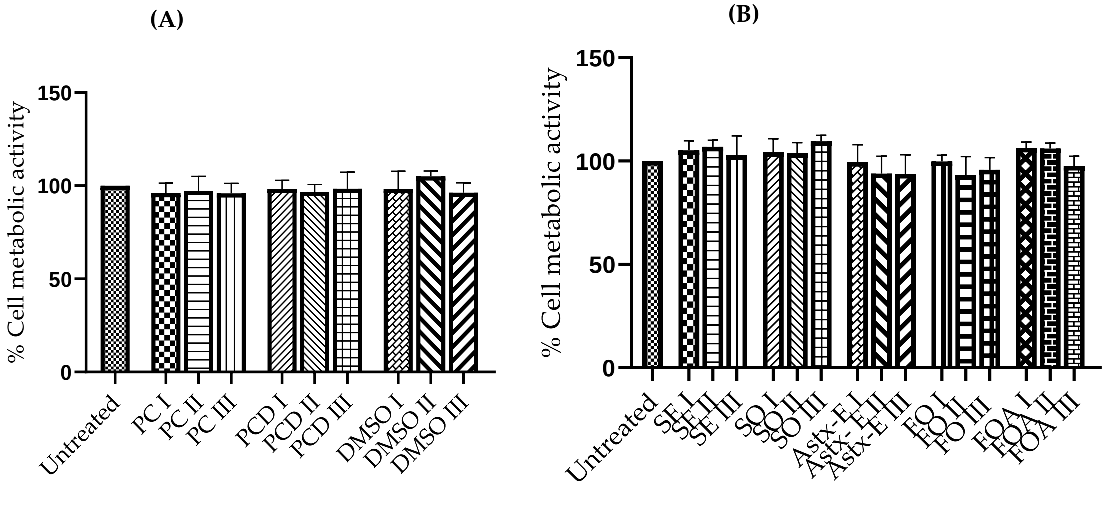


**Figure S5.** Cell metabolic activity of 3T3-L1 preadipocytes treated with various concentrations of treatments and vehicles. Cells were treated with various concentrations of treatments, and the vehicles, as explained in the methods section. (**A**) Vehicles: PC, DMSO, PCD; (**B**) Treatments: SE, SO, Astx-E, FO, and FO+Astx-E. Data were analyzed using one-way ANOVA to determine significance ($P<0.05$). Values are expressed as means ± SD, n=3. Untreated=Untreated cells, PC=L-$\alpha$-phosphatidylcholine (I, II, III represents 15, 30, and 60 μg/mL culture medium, respectively), PCD=PC+DMSO [(I, II, III represents 15 μg + 0.03%, 30 μg + 0.06%, 60 μg + 0.1%)/mL culture medium, respectively], DMSO=dimethyl sulfoxide (I, II, III represents 0.03, 0.06, and 0.1%, respectively), SE=shrimp extract (I, II, III represents 0.125, 0.25, and 0.5 mg/mL culture medium, respectively), SO=shrimp oil (I, II, III represents 0.125, 0.25, 0.5 mg/mL culture medium, respectively), Astx-E=esterified astaxanthin (I, II, III represents 7.95, 15.9, and 31.8 ng/mL culture medium, respectively), FO=fish oil (I, II, III represents 0.125, 0.25, and 0.5 mg/mL culture medium, respectively), FOA= FO+Astx-E [(I, II, III represents 0.125 mg + 7.95 ng, 0.25 mg + 15.9 ng, 0.5 mg + 31.8 ng )/mL culture medium, respectively].

**Supplementary S6**
**Primer sequences for Real-time quantitative polymerase chain reaction**

**Table S1.** Primer sequences for Real-time quantitative polymerase chain reaction

| Gene | Sequence | | Ascension No |
|---|---|---|---|
| ***Pparγ*** | 5′-GAGCTGACCCAATGGTTGCTG-3′ | Forward | XM_017321456.1 |
| | 5′-GCTTCAATCGGATGGTTCTTC-3′ | Reverse | |
| ***Srebp1c*** | 5′-CGGCTCTGGAACAGACACTG-3′ | Forward | NM_001313979.1 |
| | 5′-TGAGCTGGAGCATGTCTTCG-3′ | Reverse | |
| ***Scd1*** | 5′-CACCTGCCTCTTCGGGATTT-3′ | Forward | NM_009127.4 |
| | 5′-CTTGACAGCCGGGTGTTTG-3′ | Reverse | |
| ***Dgat2*** | 5′-CTGCTGTTGGCTGGTTTCAC-3′ | Forward | NM_026384.3 |
| | 5′-CAGGAGGATATGCGCCAGAG-3′ | Reverse | |
| ***Fasn*** | 5′-CTGCGGAAACTTCAGGAAATG-3′ | Forward | NM_007988.3 |
| | 5′-GGTTCGGAATGCTATCCAGG-3′ | Reverse | |
| ***Glut-4*** | 5′-GATTCTGCTGCCCTTCTGTC-3′ | Forward | AB_008453.1 |
| | 5′-ATTGGACGCTCTCTCTCCAA-3′ | Reverse | |
| ***Rplp0*** | 5′-AATTTCAATGGTGCCTCTGG-3′ | Forward | NM_007475.5 |
| | 5′-TCACTGTGCCAGCTCAGAAC-3′ | Reverse | |

Primers used in qPCR were designed using NCBI primer blast and purchased from IDT Technologies. Pparγ: peroxisome proliferator-activated receptor-gamma; Srebp1c: sterol regulatory element-binding protein 1; Scd1: stearoyl-CoA desaturase 1; Dgat2: diacylglycerol O-acyltransferase 2; Fasn: fatty acid synthase; Glut-4: Glucose transporter type 4; Rplp0: ribosomal protein large.
